# Phage Therapy as a Focused Management Strategy in Aquaculture

**DOI:** 10.3390/ijms221910436

**Published:** 2021-09-28

**Authors:** José Ramos-Vivas, Joshua Superio, Jorge Galindo-Villegas, Félix Acosta

**Affiliations:** 1 Grupo de Investigación en Acuicultura, Universidad de Las Palmas de Gran Canaria, 35214 Las Palmas de Gran Canaria, Spain; joseramosvivas@gmail.com (J.R.-V.); felix.acosta@ulpgc.es (F.A.); 2 Research Group on Foods, Nutritional Biochemistry and Health, Universidad Europea del Atlántico, 39011 Santander, Spain; 3 Department of Project Management, Universidad Internacional Iberoamericana, Campeche 24560, Mexico; 4 Faculty of Biosciences and Aquaculture, Nord University, 8049 Bodø, Norway; joshua.superio@nord.no

**Keywords:** aquaculture, bacteriophages, disease management, fish, immunology, lytic enzymes, pathogens

## Abstract

Therapeutic bacteriophages, commonly called as phages, are a promising potential alternative to antibiotics in the management of bacterial infections of a wide range of organisms including cultured fish. Their natural immunogenicity often induces the modulation of a variated collection of immune responses within several types of immunocytes while promoting specific mechanisms of bacterial clearance. However, to achieve standardized treatments at the practical level and avoid possible side effects in cultivated fish, several improvements in the understanding of their biology and the associated genomes are required. Interestingly, a particular feature with therapeutic potential among all phages is the production of lytic enzymes. The use of such enzymes against human and livestock pathogens has already provided in vitro and in vivo promissory results. So far, the best-understood phages utilized to fight against either Gram-negative or Gram-positive bacterial species in fish culture are mainly restricted to the *Myoviridae* and *Podoviridae*, and the *Siphoviridae*, respectively. However, the current functional use of phages against bacterial pathogens of cultured fish is still in its infancy. Based on the available data, in this review, we summarize the current knowledge about phage, identify gaps, and provide insights into the possible bacterial control strategies they might represent for managing aquaculture-related bacterial diseases.

## 1. Phage Biology and Spatial Distribution

Bacteriophages or phages, in short, are an alternative to antimicrobials to fight against bacteria due to their unique host range that provides them with an excellent specificity. In addition, contrary to the antibiotic’s negative physiological effects on the host and the generation of bacterial resistance, the use of phages is eco-friendly and without major drawbacks [1,2]. Besides, phages produce lytic enzymes with the ability to act directly on the bacterial cell wall. An important associated advantage is that phages are ubiquitous to all fresh and saltwater environments representing a virtually unlimited source of virions and lytic enzymes. In seawater, the number and variety of phages have a direct and crucial impact on the variability of microbial communities which directly modulate the global biogeochemical cycles in the oceans [3,4]. Quantitative analyses of marine waters using transmission electron microscopy demonstrated that non-tailed viruses are the most abundant, followed by tailed viruses of the families *Myoviridae* and *Podoviridae* [5]. This example represents a huge gene reservoir across Earth’s ecosystems. Despite the great awakening interest in phage therapy and the discovery of a vast reservoir of new genes available in the phages of aquatic ecosystems, the composition the phage populations in the different fish species in aquaculture, either from freshwater or saltwater environments are not yet fully understood. 

## 2. Phage’s Life Cycle

**Figure 1:**
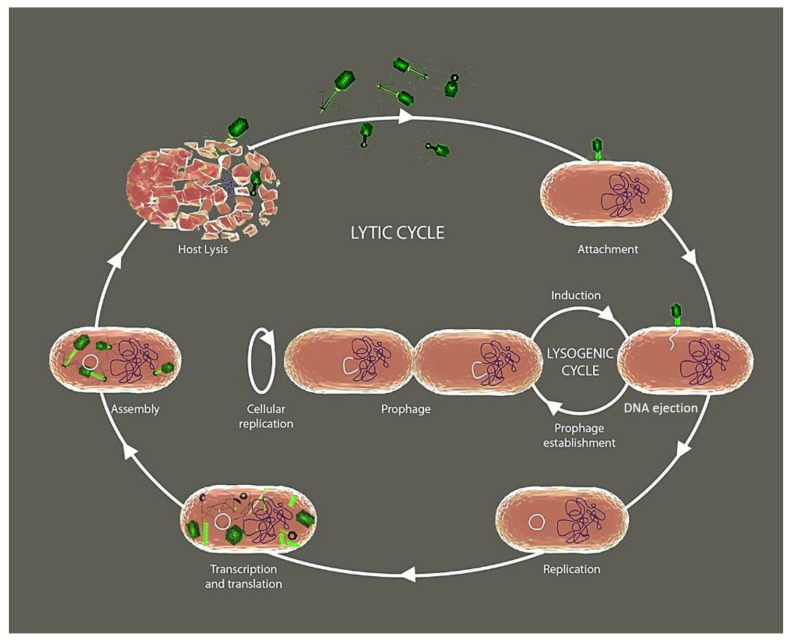
The lytic and lysogenic cycle of bacteriophages. The lytic cycle comprises a series of events from attachment of the bacteriophage to the bacterial cell membrane, to the release of daughter phages by the destruction of its bacterial host. In the lysogenic cycle, phage DNA integrates into the bacterial genome without major consequences for the bacterial cell, and where the nucleic acid of the virus replicates along with that of its host.

The phages like any other viruses depend on the metabolism of their bacterial host for reproduction. During the reproductive process, most phage types completely consume the resources of their host and kill them when releasing their progeny [6]. Initially, phages must infect their host bacteria through the binding of specific receptors that selectively sense specific components of the target bacterial cell wall such as the lipopolysaccharide in Gram-negative, or peptidoglycan in Gram-positive, capsular polysaccharides, and superficial appendages such as pili and flagella [7,8,9]. Following the classical viral reproductive strategies, once the phage inserts their nucleic acid into the bacterium’s cytoplasm, the host cellular machinery is highjacked to induce extensive replication through the lytic cycle (Figure 1). Alternatively, a phage also has the capacity to insert its genetic information into the genome of the host bacterium, thus becoming a prophage. The process of prophage incorporation into the host chromosome is called lysogenization, and the resulting bacterium with the prophage is called a lysogen. Therefore, the genetic material of the prophage is transferred to each daughter cell through cell division following the lysogenic cycle (Figure 1). A huge advantage associated with the lysogenic cycle is that daughter cells will not produce new virus particles until conditions are favorable for the virus or some external stimuli stress the cell and activate the highjacked genes. An additional less known phage reproductive cycle is the so-called pseudo-lysogenic. In the pseudo-lysogenic type, the information encoded by the genome of the phage is not translated immediately, perhaps due to the lack of nutrients and energy for the bacterium. However, it remains inactive inside the host, waiting until the optimal conditions recover for the bacterium to restart its metabolic processes. Then, the phage has the capacity to start again performing the lytic or lysogenic life cycles [10]. 

## 3. Phage Lytic Enzymes and Depolymerases

With increasing development and with some of our hopes against antibiotic resistance placed on the use of complete phage particles of one or more types of phages (phage cocktails), the lytic enzymes of these viruses have begun to enter the race of clinical development [11,12]. Due to the possible break that legislation in human medicine may imply, researchers in various fields such as veterinary medicine or food science and technology have also begun to develop and produce these phage products [13,14,15]. Phage-encoded proteins with potential application in these different fields are divided into endolysins, exolysins, and depolymerases. Lysins derived from phages degrade bacterial peptidoglycans and are classified into five groups, depending on the bonds these enzymatic proteins cleave in the bacterial peptidoglycan [16]. Although their function is exclusively to degrade the cell wall of bacteria, the lytic enzymes of phages present a tremendous structural diversity and a significant number of different mechanisms of action [17,18,19,20].

A phage’s efficiency and the host’s specificity are determined by protein domains and their associated specific enzymatic activities. Their structure target and degrade the cell wall of Gram-positive or Gram-negative bacteria depending on whether they contain an enzymatic catalytic domain and a cell wall binding domain or a single catalytic domain, respectively. Most phage endolysins degrade the cell wall from inside the bacteria when the lytic cycle of the phages that grow in the bacterial cytoplasm mature [20]. However, there are also phage enzymes that degrade the bacterial cell wall from the outside; these proteins called—exolysins -or ectolysins—are of great interest for their application as antimicrobial agents because they can reach bacteria more quickly than if they must do so after a cycle of phage infection [21,22,23]. These proteins with enzymatic activity have also been used to destroy preformed biofilms by Gram-positive or Gram-negative bacteria, although most studies have been carried out in vitro [24,25,26]. Moreover, while some naturally occurring lysins have potent in vitro activity, they can be engineered to be more effective in binding or traversing the bacterial wall [27,28,29,30,31,32]. Although not well known as lysins, phage enzymes with depolymerase activity—or depolymerases—can be of great interest for fighting pathogens and the biofilms that they form since a critical component of the biofilm matrix is usually polymers of a different nature [33,34,35].

In general, lysins are more likely to lyse Gram-positive bacteria because their cell wall peptidoglycan is directly exposed on the cell surface unlike Gram-negative bacteria. However, the study of phages or their lysins has been limited to a few fish pathogens such as *Streptococcus agalactiae*, *Lactococcus garvieae*, *Renibacterium salmoninarum*, *Streptococcus iniae*, and *S. dysgalactiae*, which are highly associated with disease outbreaks in fish farms.

## 4. Interactions between Phage and the Fish Immune System

Contrary to the accomplishments garnered so far in mammals and cultured cells; limited studies have correlated the immune responses of cultured fish treatment with phages. Therefore, many knowledge gaps exist on the enhancement and link of phage activity and the fish immune system. However, the administration route of phages is perceived as crucial among the known interactions between phages and fish’s immune system [36]. In vitro studies with cultured cells obtained from mucosal surfaces and in vivo challenged experiments in fish by immersion have demonstrated that increased phage abundance in the mucus layer protects the underlying epithelium from bacterial infection [37,38]. Thus, the experimental results highlight that the immersion route enables phages to penetrate fish tissues [39]. Once inside the fish body, phages will first interact and overcome the cells of the mucosal innate immune system before searching for their bacterial prey.

Interestingly, it has been reported that phages lack an outer lipid layer, which is a typical target for the complement. Which leads to an intriguing question on how the phages evade adaptive and innate immunity elements like antibodies and phagocytes respectively to gain access to the eukaryotic cells. Transcytosis is a partial explanation which includes free uptake by endocytosis or crossing via a leaky gut allowing for passive transit through the epithelium. A less understood, but exciting mechanism exists that is regarded as the trojan horse. This mechanism explains the endocytosis of the bacterial host once colonized by the phage and is hidden inside to secure access free of recognition.

*Caudovirales* phages from the families *Myoviridae*, *Siphoviridae*, and *Podoviridae* have the ability to translocate across epithelial and endothelial cell lines [40]. Interestingly, the same three phage families reported are those commonly observed interacting with bacterial fish pathogens (See Table 1). Once phages have gained access to the fish mucosal tissue, the extra- and intra-cellular immune recognition mechanisms exert a systematic screening process [41]. However, the endocytic recognition starts only after lysosomal degradation occurs inside the cell, where pathogen recognition is mediated by classical conserved pattern recognition receptors (PRR). Some of the significant PRR responsible for sensing viral RNA identified so far include the retinoic acid inducible gene I (RIG-I) like receptors (RLRs) and several endosomal-associated TLRs, for example, TLR3, TLR7, TLR8, and TLR9 [42].

After these initial encounters with the fish tissue, phages can stimulate and modulate the host’s innate immune response [43,44,45,46,47]. Several intracellular or facultative intracellular bacterial pathogens have been reported to trigger immune responses in cultured fish. Among them are *Photobacterium damselae* subsp. *piscicida*, *Renibacterium salmoninarum*, *Piscirikettsia salmonis*, *Edwardsiella tarda*, *Edwardsiella ictaluri*, *Yersinia ruckeri*, and *Vibrio parahaemolyticus* were identified. However, studies on fish immunity showing the detailed interaction of members in the genus *Edwardsiella* and *Vibrio* with phages have rarely been reported.

### 4.1. Phage-Mediated Activation of Inflammation

Bacteriophage treatment was associated with opposite shifts in the inflammatory response in several test models, both in vivo and in vitro [48,49,50,51]. However, the results seem to depend not only on the cellular or animal model used but also on the type of phage applied and the panel of cytokines analyzed. Phage therapy in humans can also modify the levels of some cytokines produced by blood cells in treated patients [39]. In fish, some researchers have analyzed the cytokines’ response to the presence of bacteriophages alone or the coinfection of phages with their target bacteria. For example, phage therapy reduced the expression of the proinflammatory cytokines *tnfa* and *il1b* in the inflammatory response generated by *Pseudomonas aeruginosa* infection in zebrafish embryos [52,53]. Besides, using the adult zebrafish (*Danio rerio*) and the *E. tarda* model of infection, other authors also showed that although a phage treatment induced the expression of cytokine genes at specific time points, a robust proinflammatory response was undetected in the host [54]. Furthermore, a recent study has shown that a phage lysate of *A. hydrophila* induced a more robust immune response in *Cyprinus carpio* when compared to a formalin killed vaccine [55]. As a proof-of-concept, a novel commercial preparation containing three bacterial phages (BAFADOR^®^) applied on European eel (*Anguilla anguilla*) caused the stimulation of cellular and humoral immune parameters in response to an experimental challenge with *A. hydrophila* and *P. fluorecense* [56].

### 4.2. Phage-Specific Adaptive Responses

Due to the protein structure of the phage envelope, these proteins are the target of the adaptive immune system, which response with the production of neutralizing antibodies against them. Early studies with mice and even amphibians showed that phage exposure of the animals induced primary and secondary antibody responses [57,58,59]. It is expected that some phage epitopes stimulate an antibody response in experimental models. However, antibody production depends on the route of phage administration, the application schedule and dose, and individual features of a phage. Consequently, the results of studies where an antibody response to phages has been verified are very heterogeneous. Phagocytosis by immune patrolling cells seems to be a significant process of bacteriophage neutralization within animal bodies [60]. Moreover, although blood in humans and animals, including fish, is deemed sterile, genomic analysis has shown a rich phage community, which inevitably comes into continuous contact with immune cells in this rich fluid [47]. Despite these mechanisms of phagocytosis, antigen presentation, and antibody production by the immune cells against phages, the number of antibodies produced does not affect phage therapy outcomes.

On the other hand, due to the numerous and constant presence of large numbers of phages in our microbiota, it is not surprising that a low but stable background of antibodies against them is produced. Therefore, in some human or animal tests, high antibody levels have not been found against the phages used. Phage-derived RNA and ssDNA could directly contribute to B cell activation and the synthesis of anti-bacteriophage antibodies [61,62]. Despite the production of antibodies by animals against phage core or tail proteins, the induction of antibodies seems irrelevant for treating infections because the antibacterial effects of phages are faster than antibody formation in acute infections [63]. Conversely, the production of antibodies against phages could interfere with the outcome of the infection in chronic infections [64]. However, no robust studies have demonstrated an antibody-mediated immune response after inoculation or experimental infection with phages in fish.

## 5. Phage Diversity in the Aquatic Environment

Bacteriophages or phages are relatively simple viruses that target a bacterial host. These viruses are extraordinarily abundant and diverse. Phages are common in soil and human and animal guts, so they are readily isolated from feces and sewage. Moreover, due to the exponential advancement of bioinformatics and massive sequencing techniques, the geospatial distribution of viruses in freshwater or saltwater ecosystems is an incipient area of research. Fish and shellfish that inhabit those environments also harbor copious amounts of phages in their digestive tracts [65,66]. Bacteriophages floating in waters are also very abundant, with an estimate of 10 million virus-like particles in 1 mL of seawater [67]. 

Virus classification is typically based on characteristics such as type of nucleic acid, virion structure or morphology, replication mode, host target and clinical and epidemiological features of disease they cause, and even geographical distribution [68]. Researchers working in aquaculture mainly use Transmission Electron Microscopy (TEM) and nucleic acid enzymatic sensitivity to determine the morphology and nucleic acid type of the phages used. In recent years, thanks to cryo-Electron Microscopy (cryo-EM), Nuclear Magnetic Resonance (NMR), and X-ray crystallography analysis, we have been able to study the structures of numerous bacteriophages. Genomic sequencing and analysis of the DNA isolated from phages can also provide reasonably accurate taxonomic information. The use of sequences alone sets a significant challenge for traditional phage taxonomy; historically it has been based primarily on descriptive definitions, but nothing prevents these techniques from being used simultaneously for the better characterization and study of phages. 

There are three primary basic morphologies in bacteriophages: (1) filamentous form, (2) icosahedral form, and (3) icosahedral form with a tail through which it binds to its target bacteria. The latter is the best known and most studied morphology, with three components: a capsid where the dsDNA genome is packed, a tail—which can be rigid or flexible, short, or long—that serves as a syringe during infection to transfer the phage genome into the bacterial cell, and a unique recognition system at the end of the tail that specifically recognizes the host cell and penetrates its cell wall.

As temperate phages are expected to execute both types of infection modes, only lytic phages can be used to fight bacterial infections because their action destroys the host and causes new nascent phages to spread among adjacent bacterial populations. Phages showing the lytic mechanism of replication are also known as virulent phages, while phages exhibiting lytic and lysogenic cycles are temperate phages. The temperate phages exhibit horizontal gene transfer, where they can transfer antibiotic resistance genes to other bacteria, and therefore they are not preferred for phage therapy unless their genome is analyzed, and it is shown that they do not contain those resistance genes

In recent years, numerous bacteriophages that target the primary bacterial fish pathogens have been isolated and partially characterized (Table 1). Many of these studies with phages have proven their efficiency against pathogenic bacteria in vitro. In fact, some researchers have even carried out field trials over the last decade using different routes of administration (orally, intraperitoneally, or intramuscularly injected, administered in feed, or added to fish thank water). Most of these studies use the work of Ackermann or the criteria of the International Committee on Taxonomy of Viruses (ICTV, accessible online) as a basis to identify their phage isolates [69,70,71]. For further taxonomic classification and phage characterization, more detailed information, such as genomic data, has begun to be included in scientific publications [72,73,74].

As phages are ubiquitously present in nature, the sources of isolated phages against fish pathogens are quite diverse (Table 1). Fish can harbor phages easily as demonstrated long ago in an experiment in which phages were detected in fish tissue after bath exposure to a specified concentration of an *E. coli* phage [39]. Detection of bacteriophages can be carried out by classical infection assays such as single-host, double or multiple-host enrichment, followed by selection through phage plating with the target host strain, allowing the determination of phage infectivity. As observed in Table 1 and Table 2, most researchers working with pathogenic fish bacteria prefer to use enrichment with a single strain to isolate phages. Instead of enriching phage-containing samples, some authors use the phage isolation technique called the double-agar layer [75,76], choosing a single indicator strain.

After phage amplification from their initial host strain, researchers use phage DNA extraction, DNA sensitivity to DNases or RNases, and virion microscopy visualization for quick taxonomic classification. Bacterial whole-genome sequencing (WGS), which focuses on the prophage state inside the bacterial chromosome, is also helpful for knowing the whole phage coding sequences and phage viability determination. Bacteria can carry more than half a dozen complete or incomplete prophages on their chromosomes. Therefore, researchers can use genomic characterization to choose lytic phages that do not contain enzymes that favor integration into the bacterial chromosome, such as recombinases, integrases, or repressor sequences of the lytic cycle—characteristics of lysogenic phages—and of course that do not contain resistance genes or bacterial virulence factors.

**Figure 2:**
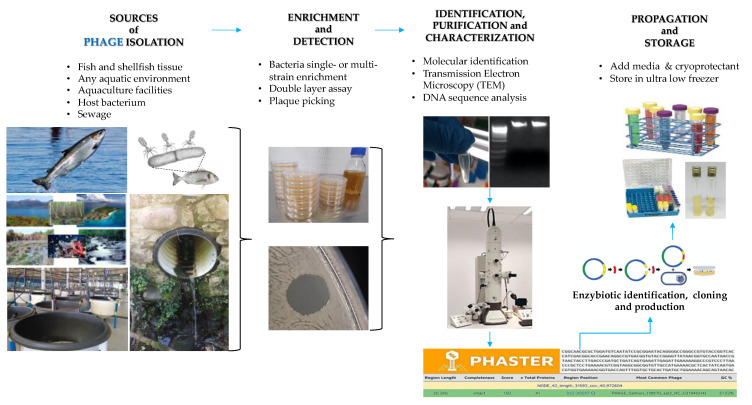
Schematic steps followed for the isolation and characterization of phages intended for use in aquaculture. From left to right: General steps for phage isolation, enrichment, detection, identification, characterization, propagation, and storage.

It is convenient to integrate as many techniques as possible in phage studies to offer more robust information regarding the phage of interest. Recently, new and more complex techniques such as Atomic Force Microscopy are able to provide information about phage integrity close to physiological conditions [77,78,79]. The preferred tool currently utilized for classifying phages is transmission electron microscopy, followed by phage DNA sequencing (Table 1). The steps followed to isolate and characterize phages used in in vitro assays or field trials against fish and shellfish bacterial pathogens are shown (Figure 2). The phages used against Gram-negative fish pathogens belong primarily to the *Myoviridae* family, followed by the *Siphoviridae* and *Podoviridae* families. Other minor families that display lytic activity against Gram-negative pathogens are Tectiviridae and *Autographiviridae*. Against Gram-positive pathogens, the *Siphoviridae* family predominates, although phages of the *Picoviridae* and *Caudoviridae* families with lytic activity against these pathogens have also been isolated.

## 6. Bacteriophages against Biofilms in Aquaculture Facilities

All bacterial fish pathogens can form biofilms in vitro and in vivo given the proper environmental conditions [80,81,82]. Biofilm formation is an intrinsic characteristic of bacteria because living within these structures favors the uptake of nutrients and protection against the physical, chemical, or biological agents found in aquatic environments. This process always occurs sequentially, and the mechanisms have been understood for a couple of decades [83]. Firstly, the adhesion of the bacteria to a living or inert surface is required. This process used to be favored both by intrinsic factors of the bacteria such as the mobility or the expression of a polysaccharide capsule [80] and by physical—chemical and environmental factors, such as temperature [81], or the type of substrate to which the bacteria attach itself. After this union, the formation of microcolonies occurs, and these small groups of bacteria will evolve into big macro-colonies that make up the biofilm. An essential characteristic of mature biofilms is that they contain a heterogeneous matrix that maintains cohesion between the bacteria that form it and favors their adherence to the surface on which the biofilm is formed. Biofilms are extremely difficult to eradicate because the bacterial cells inside them are more protected from any chemical or biological agent, so they tend to easily resist conventional antibiotics and common disinfectants [84,85]. Biofilm formation is essential in veterinary medicine since they can represent the focus of recurrent infections [86,87]. From the fully formed and mature biofilms, bacteria are released and constitute a population of planktonic cells that will form new biofilms in other places, allowing recurrent infections or the contamination of new environments favorable for their proliferation. 

Biofilms are also of particular interest in the food industry, where there are a series of ubiquitous microorganisms that are very difficult to eliminate from the food itself and food processing settings and environments. Phages alone or in phage cocktails were successfully used against both Gram-positive [88,89,90,91,92] and Gram-negative bacterial biofilms [93,94,95,96,97,98]. Despite these assays being performed in a wide variety of situations and against numerous pathogens in human medicine, veterinary medicine, and food production systems, these studies have been carried out mainly in the laboratory in vitro. Therefore, it will also be interesting to know the options that fish pathologists may have to eradicate the biofilms that bacteria produce in aquaculture. Phages were also tested against bacterial fish pathogens producing biofilms in vitro, but the number of studies conducted following these settings is very minimal [99]. Therefore, it is important that the action of the phages against fully formed biofilms is also evaluated when searching for lytic phages against pathogenic fish bacteria.

**Table 1:** Phages used against Gram-negative bacterial fish and shellfish pathogens.

Gram-Negative Targets	Source	Enrichment ^ɸ^	Characterization Method	Phage Strains Name	Family *	Genome Length	References
***Aeromonas hydrophila***	River water	No	TEM	ɸ2 and ɸ5	*Myoviridae*	~20 kb	[100]
	Fishponds; Polluted rivers	Single	TEM	N21, W3, G65, Y71 and Y81	*Myoviridae; Podoviridae*	n.d.	[101]
	Stream water	Single	TEM, dsDNA	pAh-1	*Myoviridae*	~64 kb	[102]
	Sea water	Single	TEM, DNA sequencing	Akh-2	*Siphoviridae*	114,901 bp	[103]
	Carp tissues	Single	TEM	AHP-1	*Myoviridae*	n.d.	[104]
	Lake water	Single	TEM, dsDNA, DNA sequencing	AhyVDH1	*Myxoviridae*	39,175 bp	[105]
	River water	No	TEM, dsDNA, DNA sequencing	MJG	*Podoviridae*	45,057 bp	[106]
	Sewage water	Single	TEM	AH1	n.d.	n.d.	[107]
	Striped catfish pond water	Single	TEM, dsDNA, DNA sequencing	PVN02	*Myoviridae*	51,668 bp	[108,109]
	River water		TEM, dsDNA	pAh1-C pAh6-C	*Myoviridae*	55 kb 58 kb	[110]
	Wastewater	No	TEM, dsDNA, DNA sequencing	Ahp1	*Podoviridae*	~42 kb	[111]
***Aeromonas punctata***	Stream water	Single	TEM, dsDNA	IHQ1	*Myoviridae*	25–28 kb	[112]
***Aeromonas salmonicida***	River waters, two passing through fish farms	Single	TEM, DNA sequencing	SW69-9 L9-6 Riv-10	*Myoviridae*	173,097 bp, 173,578 bp and 174,311 bp	[113]
	River water	Single	TEM, DNA sequencing	phiAS5	*Myoviridae*	225,268 bp	[114]
	Sediment of a Rainbow trout culture farm	Single	TEM, dsDNA, DNA sequencing	PAS-1	*Myoviridae*	~48 kb	[115]
	Wastewater from a seafood market	No	TEM, DNA sequencing	AsXd-1	*Siphoviridae*	39,014 bp	[116]
	Sewage network water from a lift station	Single	TEM	AS-A AS-D AS-E	*Myoviridae*	n.d.	[117,118]
	River water	No	TEM	HER 110	*Myoviridae*	n.d.	[119,120]
***Aeromonas* spp.**	Gastrointestinal content of variated fish species	No	TEM, DNA sequencing	phiA8-29	*Myoviridae*	144,974 bp	[66,121]
***Citrobacter freundii***	Sewage water	No	TEM, DNA sequencing	IME-JL8	*Siphoviridae*	49,838 bp	[122]
***Edwardsiella ictaluri***	Water from catfish ponds	Single	TEM, dsDNA, DNA sequencing	eiAU eiDWF eiMSLS	*Siphoviridae*	42.80 kbp 42.12 kbp 42.69 kbp	[123,124]
	River water	Multiple	DNA Sequencing	PEi21	*Myoviridae*	43,378 bp	[125,126]
	Striped catfish kidney and liver	Single	TEM, dsDNA	MK7	*Myoviridae*	~34 kb	[127]
***Edwardsiella tarda***	Seawater	Single	TEM, dsDNA	ETP-1	*Podoviridae*	~40 kb	[54]
	River water	No	TEM, DNA sequencing	pEt-SU	*Myoviridae*	276,734 bp	[128]
	Wastewater	Single	DNA sequencing	PETp9	*Myoviridae*	89,762 bp	[129]
	Fish tissues and rearing seawater	No	TEM, DNA sequencing	GF-2	*Myoviridae*	43,129 bp	[130]
***Flavobacterium columnare***	River water	Single	TEM, DNA sequencing	FCL-2	*Myoviridae*	47,142 bp	[38,131,132]
	Fishpond’s water and bottom sediments	No	TEM, dsDNA	FCP1-FCP9	*Podoviridae*	n.d.	[133]
***Flavobacterium psychrophilum***	Rainbow trout farm water	Single/double	TEM, dsDNA	^ø^ (FpV-1 to FpV-22)	*Podoviridae* *Siphoviridae* *Myoviridae*	(~8 to ~90 kb)	[134,135]
	Ayu kidneys and pondwater collected from ayu farms	Multiple	TEM, dsDNA	PFpW-3, PFpC-Y PFpW-6, PFpW-7 PFpW-8	*Myoviridae; Podoviridae; Siphoviridae*	n.d.	[136]
***Photobacterium damselae* subsp. *damselae***	Raw oysters	Single	TEM, dsDNA	Phda1	*Myoviridae*	35.2–39.5 kb	[137]
	Gastrointestinal tract of lollipop catshark	Single	TEM, DNA sequencing	vB_Pd_PDCC-1	*Myoviridae*	237,509 bp	[138]
***Pseudomonas plecoglossicida***	Ayu pond water and diseased fish	No	TEM, DNA sequencing	PPpW-3 PPpW-4	*Myoviridae Podoviridae*	43,564 bp 41,386 bp	[139,140]
***Pseudomonas aeruginosa***	Wastewater	No	TEM, DNA sequencing	MBL	n.d.	42,519 bp	[141]
***Shewanella* spp.**	Wastewater from a marketplace	Single	TEM, DNA sequencing	SppYZU01 to SppYZU10	*Myoviridae; Siphoviridae.*	SppYZU01 (43.567 bp) SppYZU5 (54.319 bp)	[142]
***Tenacibaculum maritimum***	Seawater	Multiple	TEM, DNA sequencing	PTm1 PTm5	*Myoviridae*	224,680 bp 226,876 bp	[143]
***Vibrio alginolyticus***	Aquaculture tank water	Single	TEM, DNA sequencing	VEN	*Podoviridae*	44,603 bp	[144]
	Marine sediment	No	TEM, DNA sequencing	ValKK3	*Myoviridae*	248,088 bp	[145]
	Marine water	Single	TEM, dsDNA	St2 Grn1	*Myoviridae*	250,485 bp 248,605 bp	[146]
***Vibrio anguillarum***	Soft tissues from clams and mussels	No	TEM, dsDNA	309 ALMED CHOED ALME CHOD CHOB	Several shapes	~47–48 kb	[147]
	Sewage water	Double	dsDNA	VP-2 VA-1	n.d.	n.d.	[148]
	Water samples from fish farms	Multiple	TEM, DNA sequencing	^ø^ H1, H7, S4-7, H4, H5 H8, H20 S4-18, 2E-1, H2	*Myoviridae Siphoviridae Podoviridae*	~194–195 kb ~50 kb ~45–51 kb	[149]
***Vibrio campbellii***	Host strain (*V. campbellii*) isolated form a dead shrimp	No	TEM, DNA sequencing	HY01	*Siphoviridae*	41.772 bp	[150]
	Hepatopancreas of Pacific white shrimp	Single	dsDNA, DNA sequencing	vB_Vc_SrVc9	*Autographiviridae*	~43.15 kb	[151]
***Vibrio harveyi***	Shrimp farm, hatcheries and marine water	Multiple	TEM, dsDNA	A	*Siphoviridae*	n.d.	[152]
	*Vibrio harveyi*	No	TEM, dsDNA	VHML	*Myovirus*-like	n.d.	[153]
	Shrimp pond water	Single	TEM, dsDNA	PW2	*Siphoviridae*	~46 kb	[154]
	Water and sediment samples	Single	TEM, dsDNA	VHM1, VHM2 VHS1	*Myoviridae*, *Siphoviridae*	~55 kb, ~66 kb ~69 kb	[155]
	Hatchery water and oyster tissues	Single	TEM, dsDNA	vB_VhaS-a vB_VhaS-tm	*Siphoviridae*	~82 kb ~59 kb	[156]
	Commercial clam samples	Multiple	Genomic analysis, dsDNA	^ø^ VhCCS-01 VhCCS-02 VhCCS-04 **VhCCS-06** VhCCS-17 VhCCS-20 VhCCS-19 VhCCS-21	*Siphoviridae*, *Myoviridae*	n.d.	[157]
	Oyster, clam, shrimp, and seawater samples	No	TEM, DNA sequencing	VHP6b	*Siphoviridae*	78,081 bp	[158]
	shrimp hatchery and farm water, oysters from estuaries, coastal sea water	Multiple	TEM, dsDNA	Viha10 Viha8 Viha9 Viha11 Viha1 to Viha7	*Siphoviridae* - *Siphoviridae* *Myoviridae* (Viha4)	n.d. ~44–94 kb ~85 kb (Viha4)	[159,160]
	Seawater sample	Single	TEM	VhKM4	*Myoviridae*	n.d.	[161]
***Vibrio ordalii***	Macerated specimens of mussels	No	TEM, DNA sequencing	B_VorS-PVo5	*Siphoviridae*	80,578 bp	[162]
***Vibrio parahaemolyticus***	Sewage sample	No	TEM, dsDNA	VPp1	*Tectiviridae*	~15 kb	[163]
	Polluted seawater	No	TEM, dsDNA	KVP40 KVP41	*Myoviridae*	n.d.	[164,165]
	Seawater or mussels	Single	dsDNA	SPA2 SPA3	n.d.	~21 kb	[166]
	Coastal water	Single	TEM, DNA sequencing	pVP-1	*Siphoviridae*	111,506 bp	[167,168]
	*V. parahaemolyticus* isolated from sewage samples collected from an aquatic product market	No	TEM, DNA sequencing	vB_VpS_BA3 vB_VpS_CA8	*Siphoviridae*	58,648 bp 58,480 bp	[169]
	Shrimp pond water	Single	TEM, DNA sequencing	VP-1	*Myoviridae*	150,764 bp	[170]
	Coastal sand sediment	double	TEM, DNA sequencing	VpKK5	*Siphoviridae*	56,637 bp	[171,172]
***Vibrio splendidus***	Raw sewage obtained from local hatcheries	Single	TEM	PVS-1, PVS-2 PVS-3	*Myoviridae; Siphoviridae*	n.d.	[173]
	Seawater near a fish farm cage	Single	TEM, DNA sequencing	vB_VspP_pVa5	*Podoviridae*	78,145 bp	[174]
***Vibrio coralliilyticus***	sewage in oyster hatchery	Single	TEM	pVco-14	*Siphoviridae*	n.d.	[175]
***Vibrio vulnificus***	Seawater sample	Single	TEM, DNA sequencing	SSP002	*Siphoviridae*	76,350 bp	[176,177]
	Abalone samples	No	TEM, sequencing	VVPoo1	*Siphoviridae*	76,423 bp	[178]
	Initial host strain (*V. vulnificus*)	No	TEM	VV1 VV2 VV3 VV4	*Tectiviridae*	n.d.	[179]
***Vibrio* sp.**	Sewage draining exits	Single	TEM, DNA sequencing	VspDsh-1 VpaJT-1 ValLY-3 ValSw4-1 VspSw-1	*Siphoviridae*	46,692 bp 60,177 bp 76,310 bp 79,545 bp 113,778 bp	[180]
***Yersinia ruckeri***	Wastewater containing suspended trout feces from a settling pond at a trout farm	Single	TEM	NC10	*Podoviridae*	n.d.	[181]
	Sewage	No	TEM	YerA41 (several phages)	icosahedral head, contractile tail	n.d.	[182]
	Sewage	No	TEM, DNA sequencing, dsDNA	R1-37	*Myoviridae*	~270 kb	[183,184]

^ɸ^ Phage enrichment with “single” or “multiple” bacterial hosts; * Classification determined by the authors; TEM (Transmission Electron Microscopy); dsDNA (Double stranded DNA); n.d. (Not determined); ^ø^ Several phage strains were isolated but only selected strains were fully characterized.

**Table 2:** Phages used against Gram-positive bacterial fish and shellfish pathogens.

Gram-Positive Targets	Source	Enrichment ^ɸ^	Characterization Method	Phage Strains Name	Family *	Genome Length	References
***Lactococcus garvieae***	*L. garvieae* isolated from diseased yellowtail	No	TEM, dsDNA	PLgY(16)	*Siphoviridae*	n.d.	[185]
	Yellowtail (Y) Water (W) Sediments (S)	Single	TEM, dsDNA	PLgW1-6 PLgY16 PLgY30 PLgY886 PLgS1	*Siphoviridae*	>20 kbp	[186,187,188]
	Domestic compost	Single	TEM, DNA sequencing	GE1	*Siphoviridae*	24,847 bp	[189]
	*L. garvieae* host	No	TEM, DNA sequencing	PLgT-1	*Siphoviridae*	29,284 bp	[190,191,192]
	Rainbow trout farm water	Single	TEM, DNA sequencing	WP-2	*Picovirinae*	18,899 bp	[193]
***Streptococcus agalactiae***	Tilapia pond	No	TEM	HN48	*Caudoviridae*	n.d.	[194]
***S. iniae***	*S. iniae* host	No	TEM, dsDNA	vB_SinS-44 vB_SinS-45 vB_SinS-46 vB_SinS-48	*Siphoviridae*	~51.7 kb ~28.4 kb ~66.3 kb ~27.5 kb	[195]
***Weissella ceti***	*W. ceti* host strain	No	TEM	PWc	*Siphoviridae*	38,783 bp	[196]

^ɸ^ Phage enrichment with “single” or “multiple” bacterial hosts; * Classification determined by the authors; TEM (Transmission Electron Microscopy); dsDNA (Double stranded DNA); n.d. (Not determined).

## 7. Potential of Phage Therapy in Aquaculture Settings

During the fish and shellfish production cycle, these animals are already in daily contact with billions of bacteriophages, which assures us that they are safe. However, in their use against bacterial infections where massive phage production is required, we must consider several factors. 

As phage treatments constantly require isolating the bacterium causing the disease, once a helpful phage is characterized against this bacterial strain, a stable batch of technically challenging preparations must be produced for field use. Consequently, one of the most critical challenge for microbiologists working directly or indirectly with aquaculture is the standardization of stocks used to treat infections or combat biofilms in aquaculture facilities. These stocks require strict quality control for purity, viability, and stability, implying that the correct conservation of the stocks is necessary for preparations containing single or mixed phages (phage cocktail). Titer, dosage, and quality of phage preparations are crucial parameters in standardizing experiments in the laboratory and experimental infections in field trials. Since we know that while some phages can grow exponentially inside a bacterial population from a low initial concentration, other phages need to maintain a relationship between the number of bacteria and the number of phage particles to achieve an adequate performance. Therefore, we must empirically verify this critical parameter. Very recently, a phage cocktail containing seven bacteriophages (three against *A. hydrophila* and four against *P. fluorescens*) has been tested in the European eel (*Anguilla anguilla*) and rainbow trout (*Oncorhynchus mykiss*), reducing the mortality of fish challenged with strains of these two species of bacteria [56,197]. Cocktails have also been used successfully in laboratory tests or small field trials in food protection or veterinary and human medicine [198,199,200,201]. In these and other studies, many phages (cocktail) are used to carry out the experiments, but in most cases, only the phage that has presented better results in vitro is subsequently characterized [117,118,133,202]. Second, it would be desirable to know phage genetics with sufficient precision. After all, we must consider that when we intend to use bacteriophages in aquaculture, they may contain genes for resistance to antibiotics or bacterial virulence genes that can produce noticeable side effects because they replicate exponentially in contact with their target bacteria. We must also remember that many antibiotic residues end up in continental or oceanic waters due to anthropogenic activities. Therefore, we must be aware that even phages isolated from aquatic environments can carry antibiotic resistance genes or virulence factors [203,204]. At present, although each time their number increases, not all phages used in in vitro or in vivo assays against fish or shellfish bacterial pathogens have been entirely genetically analyzed or characterized (Table 1 and Table 2).

The list of species of fish bacterial pathogens in which lytic phages have been studied is not complete. It may be essential to conduct these studies in species of greater interest in aquaculture, such as *Photobacterium damselae* subsp. *piscicida*, bacterial anaerobes, mycobacteria, *Nocardia*, several *Aeromonas* species, *Enterobacterales*, pseudomonads, vibrios, and the Gram-positive bacteria mentioned above. Few studies with fish bacterial pathogens have characterized or evaluated the presence or evolution of phage-resistant strains. Some works have investigated this phenomenon in various fish pathogens such as *Flavobacterium* [205,206,207], *Yersinia ruckeri* [181], *Aeromonas salmonicida* [117,208], and *Vibrio anguillarum* [148]. The mechanisms by which bacteria become resistant to phages is also an area of intensive research, especially since the discovery and application of the clustered regularly interspaced short palindromic repeats (CRISPR) system.

Most of the studies with fish pathogens have used controlled laboratory conditions to verify the control exerted by these lytic phages to their pathogenic bacterial host. However, more studies on these interactions under natural conditions would be desirable. One of the critical parameters is the multiplicity of infection (MOI). The use of high or low multiplicities of infection seems to be a key parameter for achieving effective lysis of the bacterial population and the appearance of resistance to the phages used. Therefore, comparative studies are needed to relate MOIs used in vitro and in aquatic environments, where phages are exposed to environmental conditions and factors such as dilution or variability of the target bacteria in their natural environment. A better understanding of the biology of viruses and a greater capacity to standardize the settings related to preclinical or laboratory research can also help in the advancement of regulatory affairs. As bacteriophage research continues to grow, we believe that microbiologists and immunologists working in areas related to aquaculture can use phages or their lytic enzymes to offer many promising advances in the fight against pathogenic bacterial species affecting cultured fish and shellfish.

## 8. Future Perspectives

Innovative technologies to enhance fish health and decrease diseases are paramount to achieve the global perspectives on food sustainability required by the blue growth philosophy and fulfill the blue economy goals proposed by the new aquaculture 4.0 strategy. However, the growing food demands strongly stimulate the intensification of production, generating the need for economically viable and environmentally sustainable practices that may rely on improved health management strategies. The use of antibiotics and chemotherapies in aquaculture has been widely used to reduce infectious diseases and promote growth [209]. However, since antibiotics lead to bacterial resistance generation, their use is discouraged nowadays, and several countries with major fish-producing facilities have banned their inclusion in biosecurity plans. Therefore, to improve fish robustness in aquaculture settings, several strategies based on the use of alternative sources like phytogenics [210], live microbes [211,212], their metabolic products like short-chain fatty acids (SCFA) [213] and sphingolipids [214], or structural components like peptidoglycan [215] or B-glucan [216] have been extensively tested in the last century as possible therapeutics for fish. Nevertheless, despite the entire group being potentially effective, all the previous strategies lack any specificity against a particular fish disease. 

In contrast, as discussed in the present review, phages are extremely specific in their ability to infect and destroy certain species or strains of bacteria without affecting the core commensal microbiota of the host. Therefore, phage therapy is well suited to be part of the multidimensional strategies focused on increasing fish health in culture and, at the same time, a promising tool in counteracting the rise of antibiotic-resistant bacteria. However, it is necessary to exercise caution since the potential evolution of phage resistance also exists [217]. Although resistance may occur, the concept of phage training for therapy through experimental coevolution has recently emerged and deserved future attention. Both Gram-negative and Gram-positive bacteria have been efficiently lysed by specific phages in vitro [218,219]. Therefore, the putative use of phage lytic enzymes and their interaction with the immune system of cultured aquatic organisms is an entirely new area demanding tremendous exploration. Although, the constant discovery of new phages with variated phenotypes and genotypes [220,221], hampers the integrative knowledge that requires stronger in vivo evidence to consolidate the specific phage-pathogenic bacteria as established biotechnology in aquaculture.

In the context of automation, recent researchers utilizing diverse mammalian models have demonstrated novel systems for single-virion identification of common pathogens using machine learning (ML) algorithm training. Consequently, easy access to big genomic data recently generated is fundamental to fuel the novel ML algorithms. The combination resulting from this may lead to the generation of a fast and accurate selection of bacteriophages with the specific characteristics and properties required to lysate the specific bacterial strains affecting fish culture grounds. Finally, creating biobanks of fish bacteriophages and their products (lysins) following established legal and regulatory frameworks for safe and stable use in countries with intensive aquaculture operations requires extensive maneuvers of the regulatory authorities and the pharmaceutical industry interested in their exploitation on a large-scale setting.
